# Awareness of Cardiovascular Disease Risk Factors by Community Pharmacists in Saudi Arabia

**DOI:** 10.3390/healthcare11020151

**Published:** 2023-01-04

**Authors:** Fahad Mohammad Sulaiteen, Ibrahim Abdulaziz Al-Zaagi, Majed Sultan Alenazi, Amani Zaben Alotaibi, Tahani Aali Alghamdi, Anum Yousaf, Sheraz Ali

**Affiliations:** 1 College of Pharmacy, King Saud University, Riyadh 11451, Saudi Arabia; 2 Pharmaceutical Care Services, King Saud Medical City, Ministry of Health, Riyadh 12746, Saudi Arabia; 3 Riphah Institute of Pharmaceutical Sciences, Riphah International University, Islamabad 46000, Pakistan; 4 Menzies Institute for Medical Research, University of Tasmania, Hobart 7000, Australia

**Keywords:** community pharmacist, cardiovascular disease, prevention, risk factor

## Abstract

Background: Pharmacists in community settings are recognized as highly accessible healthcare practitioners and demonstrate a crucial role in the primary prevention of cardiovascular disease. Evidence indicates that community pharmacists can make a significant impact on controlling cardiovascular disease risk factors, particularly on hypertension. Objectives: We aimed to assess the knowledge of community pharmacists in Saudi Arabia regarding cardiovascular disease risk factors. Methods: A cross-sectional study involving community pharmacists was conducted. The knowledge of cardiovascular disease risk factors was assessed with the Heart Disease Fact Questionnaire (HDFQ). A web link for an anonymous questionnaire was shared with the licensed community pharmacists in Saudi Arabia using the “Seha” platform of the Ministry of Health. Data analysis was performed with R version 4.0.5. Results: Three hundred seventy-four community pharmacists responded to the questionnaire. Many community pharmacists (94.4%) had satisfactory awareness of cardiovascular disease risk factors. The odds of having satisfactory HDFQ knowledge for community pharmacists seeing more than 20 individuals with diabetes per month were 20 times (AOR = 19.9, 95% CI: 1.73–260, and *p* = 0.019) more compared to those seeing fewer than 10 individuals with diabetes per month. The age of the community pharmacists and the average number of individuals with diabetes seen per month were found to be factors associated with satisfactory HDFQ knowledge. Conclusion: The practicing pharmacists had a substantial understanding of cardiovascular disease risk factors. In line with counseling and education, the implementation of community pharmacy models for improving the knowledge of pharmacists, particularly the young pharmacists, is needed to effectively assist patients with cardiovascular disease.

## 1. Introduction

Cardiovascular disease is the leading cause of mortality globally, accounting for an estimated 30% of all deaths [1]. The burden of cardiovascular disease increases due to the modern lifestyle that changed eating habits from organic to processed unhealthy food, as well as smoking and alcohol consumption [2]. In developing economies, epidemiological data on the incidence of cardiovascular disease risk factors are scarce. Cardiovascular disease can be prevented through the effective control and management of cardiovascular disease risk factors. Evidence suggests that the primary prevention of cardiovascular disease represents a cost-effective strategy and eventually reduces the burden of cardiovascular disease in the population [3]. There has been no attempt to derive a robust prevalence estimate of cardiovascular disease in Saudi Arabia, but the prevalence of chronic heart disease is 5.5% [4] and estimated to be higher in the future [5]. Moreover, many cardiac patients in Saudi Arabia possess the following risk factors: dyslipidemia, hypertension, and obesity [6]. The prevalence of dyslipidemia is between 20 and 40% [7], and of obesity, 24.7% [8]. Likewise, a recent study showed that about 31.4% of Saudis are hypertensive [6].

Patients often face difficulties in approaching primary care physicians, whereas healthcare costs are continuously increasing, which necessitates the greater integration of pharmacy professionals as providers of health services and members of the healthcare team [1,9,10,11,12]. Pharmacists working in community pharmacies are recognized as highly accessible healthcare professionals, and their role for the management of cardiovascular disease and associated risk factors is well-documented [13,14]. Further, the role of community pharmacists includes educating patients about diseases and counseling patients regarding medicines and medicines-related problems [15]. Community pharmacists improve the health of patients by reducing drug-related harms and advocating better medicine adherence, therefore preventing unnecessary hospital admissions [16].

An authoritative report in Saudi Arabia forecasted a three-fold increase in the economic burden of cardiovascular disease, from USD 3.5 billion to USD 9.8 billion, by 2035 [17]. A sedentary lifestyle and the consumption of unhealthy food are prevalent in Saudi Arabia. Likewise, recent data provide evidence that there is a rising trend of cardiovascular disease deaths across Saudi Arabia [4]. With the rising prevalence of cardiovascular disease risk factors and incidence of cardiovascular disease along with rising health care costs in Saudi Arabia, community pharmacists working in Saudi Arabia may indeed have a role in tackling the challenges pertinent to this public health issue. There is a scarcity of literature on the knowledge of cardiovascular disease among community pharmacists in Saudi Arabia. Therefore, we aimed to assess the knowledge of community pharmacists in Saudi Arabia regarding cardiovascular disease risk factors. In addition, we also determined the relationship between the knowledge of diagnostic cut-offs for common cardiovascular disease risk factors and Heart Disease Fact Questionnaire (HDFQ) score.

## 2. Material and Methods

### 2.1. Study Design and Setting

A cross-sectional study was conducted from May 2020 to March 2021. There are 8683 community pharmacies in Saudi Arabia [18]. The Ministry of Health (MOH) manages all the community pharmacies in the kingdom.

### 2.2. Data Collection

A 48-item questionnaire was adapted from the previous study, and permission was also sought from the study authors [19]. A web link for the validated English questionnaire was distributed to the licensed pharmacists working in the community pharmacies throughout Saudi Arabia. To avoid selection bias, all community pharmacists employed in 8683 community pharmacies received an invitation to participate in this study. A web link for the questionnaire was disseminated to the community pharmacists working in Saudi Arabia by using the “Seha” platform. Seha is an electronic platform that serves the health sector in Saudi Arabia by providing MOH-approved electronic services and aims to automate and unify procedures and services. Moreover, it provides electronic integration with the relevant authorities and a secure medium for the transmission of data and information [20].

The questionnaire consisted of four parts. The first part was for personal and demographic data and contained 9 questions. The second part tested the knowledge of community pharmacists about cardiovascular disease risk factors (e.g., smoking and diabetes are risk factors for developing heart disease, and high blood sugar puts a strain on the heart) using the widely validated Heart Disease Fact Questionnaire (HDFQ), a 25-item tool for assessing the knowledge of risk factors for heart disease [21,22]. The HDFQ has frequently been used in various study populations, with reliable test–retest reliability, internal consistency, and satisfactory discriminant validity [21]. The responses were true, false, or do not know. The final scores were summed up, and a total score >20 demonstrated good knowledge. The scores were also indicated in percentages. Participants with scores of less than 50% and between 50–69% showed low and moderate levels of knowledge, respectively. Participants with an HDFQ score greater than 70% demonstrated a good level of knowledge. Questions that <70% of the participants answered correctly were deemed unsatisfactory. The third part assessed the knowledge of diagnostic cut-off points for cardiovascular disease risk factors and consisted of eight questions. The questionnaire also had a brief statement describing the nature and purpose of this study and a consent part ensuring the anonymity and voluntary participation of the community pharmacists. The questionnaire was also validated among a pilot sample of community pharmacists. Completing the questionnaire took an average of 18 min. We also sent a reminder every month, via employees managing the “Seha” platform, for completing the questionnaire.

### 2.3. Sample Size and Statistical Analysis

Given an estimated community pharmacist population of 8419 [23] and based on a 95% confidence level and a 5% margin of error, we estimated an ideal sample size of 368 participants for this survey. Data analysis was performed with R version 4.0.5. Descriptive statistics was applied to report the general characteristics of the participants, correct responses of the HDFQ questionnaire, and knowledge of diagnostic cut-offs for common cardiovascular disease risk factors. Testing for normality of continuous variables was performed using the Shapiro–Wilk test. A binary logistic regression was conducted to identify factors associated with satisfactory HDFQ knowledge. A linear regression was also applied to evaluate the relationship between the knowledge of diagnostic cut-offs for common cardiovascular disease risk factors and HDFQ score. A *p*-value of <0.05 was taken as the level of significance between responses.

### 2.4. Ethical Considerations

This study was conducted according to the principles of the Declaration of Helsinki. Ethical approval was obtained from the Institutional Review Board of King Saud Medical City, prior to the initiation of this study (reference number: H1RI-06-Apr20-05). Agreeing or responding to the study questionnaire was considered as implied consent for participation.

## 3. Results

The general characteristics of the respondents are presented in Table 1. All community pharmacies received the study invitation, but only 374 community pharmacists participated in this study. About two-thirds (67%) of the respondents’ ages ranged between 25 and 34 years old. The majority (91%) of the respondents were males. About a quarter of the respondents had a Bachelor of Pharmacy qualification (73%) and less than ten years of practice as community pharmacists (75%). Sixty-two percent of the pharmacists had seen more than twenty, on average, hypertensive patients, as well as smokers, each month. Two hundred and sixty-four pharmacists (71%) claimed that they had seen over twenty patients with diabetes per month.

The results of the correct responses of the HDFQ questionnaire are given in Table 2. In 16 questions, more than 90% of the respondents correctly answered the questions. Only one-third of the pharmacists correctly responded to “men with diabetes have a higher risk of heart disease than women with diabetes.”

**Figure 1:**
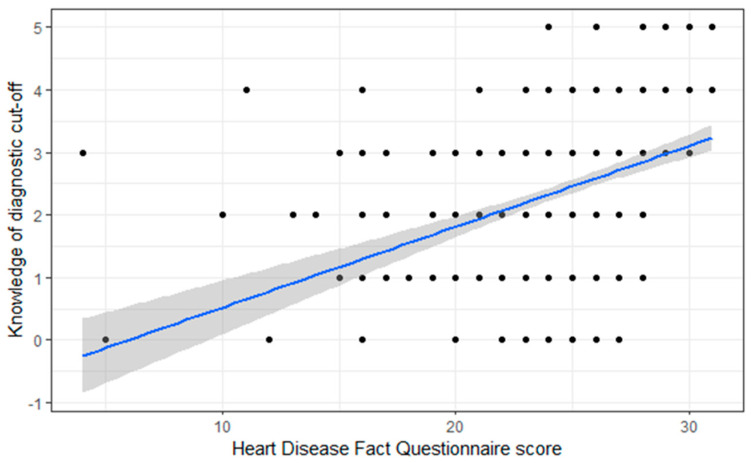
Relation between HDFQ score and knowledge of diagnostic cut-offs.

About half of the respondents correctly identified the diagnostic cut-off points for hypertension (49.2%), diabetes (46.0%), and truncal obesity in females (48%). About the knowledge of truncal obesity in males and hypercholesterolemia, 58% and 41% correctly identified the diagnostic cut-off points, respectively (Table 3). As shown in Figure 1, there was a proportional relationship between the overall HDFQ score and knowledge of diagnostic cut-offs (*p* < 0.001).

The majority (94.4%) of the pharmacists had satisfactory awareness of cardiovascular disease risk factors. The age of the respondents and average number of individuals with diabetes seen per month were found to be factors associated with satisfactory HDFQ knowledge. Accordingly, the odds of having satisfactory HDFQ knowledge for pharmacists aged 25 to 34 and 35 to 44 years old were nearly 14-fold (AOR = 13.9, 95% CI: 1.19–175, and *p* = 0.040) and 13-fold (AOR = 13.0, 95% CI: 1.19–79.1, and *p* = 0.003) more, respectively, compared to patients aged 18 to 24 years old.

The odds of having satisfactory HDFQ knowledge for community pharmacists seeing more than 20 individuals with diabetes per month were 20 times (AOR = 19.9, 95% CI: 1.73–260, and *p* = 0.019) more compared to those seeing fewer than 10 individuals with diabetes per month (Table 4).

## 4. Discussion

This study found that many pharmacists had satisfactory awareness of cardiovascular disease risk factors. Studies have demonstrated that patients have relatively lower knowledge of heart disease risks compared to healthcare professionals. A previous study reported a prevalence of 19.9% for knowledge of heart disease risks among Spanish speakers with diabetes [22]. Another study conducted in Ethiopia also reported almost half of cardiovascular disease patients had suboptimal knowledge regarding cardiovascular risk factors [24].

The findings from this study showed that the pharmacists had good knowledge on the biological and behavioral risk factors of cardiovascular disease. Additionally, the pharmacists were also majorly involved in cardiovascular disease primary prevention by lifestyle counseling and risk factors screening. Since the community pharmacists demonstrated a high level of knowledge, this finding could provide a foundation for feasibility research on the effectiveness of community pharmacy professionals in the screening of cardiovascular disease risk factors in Saudi Arabia.

This study further showed that the community pharmacists displayed good knowledge of both the behavioral and biological risk factors for cardiovascular disease. Nonetheless, in comparative terms, the knowledge of diabetes mellitus and hypercholesterolemia was lower, as only few community pharmacists correctly responded to the questions about the cut-offs for diabetes mellitus and hypercholesterolemia compared to those for hypertension. The reason for this might be due to the invasive nature of screening for diabetes mellitus (by glucometer) and hypercholesterolemia (by blood sample) that could demotivate community pharmacists as well as patients. Similarly, the finding related to diabetes was consistent with the previous report [19]. We also found a proportional relationship between the overall HDFQ score and knowledge of diagnostic cut-offs. This reflects that pharmacists with better knowledge regarding diagnostic cut-offs possess more knowledge regarding cardiovascular disease risk factors. This finding is consistent with a study performed in Nigeria [19] that reported 95.5% of the participants correctly identified cardiovascular disease risk factors.

The age of the respondents and average number of individuals with diabetes seen per month were found to be factors associated with satisfactory HDFQ knowledge. Compared to those aged less than 25 years, the older participants (25 to 45) had relatively higher odds of satisfactory knowledge of cardiovascular disease risk factors. A plausible explanation for this result may be related to work experience, as younger participants are usually new to the practice. Pharmacists providing services to more than 20 individuals with diabetes per month had higher odds of satisfactory knowledge of cardiovascular disease risk factors in comparison to those who provided services to fewer than 10 individuals with diabetes per month.

Health behavior knowledge is a crucial factor in the health belief model. The findings from this study may offer a foundation for future feasibility models focusing on the effectiveness of community pharmacists in cardiovascular disease risk screening in Saudi Arabia. It has been well-studied that the presence of cardiovascular disease risk factors is associated with higher death risk [25,26,27,28]. This risk can be managed with lifestyle and dietary counseling, which lowers the risk factors [29]. Pharmacists are, therefore, best suited to deliver this counseling because of being front-line service providers to the public. Community pharmacists could participate in the provision of education to patients via educational brochures and brief verbal discussions with each patient regarding cardiovascular disease risk factors. However, there is no specific model for the provision of education by community pharmacists in Saudi Arabia.

There are some limitations with this study. We used the HDFQ tool to assess the knowledge of pharmacists; however, this tool was initially designed for the lay public. The results reported here may be subjected to bias. A recall bias also may be introduced, as some questions relied on self-reporting.

## 5. Conclusions

The practicing pharmacists had a substantial understanding of cardiovascular disease risk factors. In line with counseling and education, the implementation of community pharmacy models for improving the knowledge of pharmacists, particularly the young pharmacists, is needed to effectively assist patients with cardiovascular disease. Pharmacists have updated courses or seminars, so that the variable of whether “they see or not” patients with a certain pathology results in being more up-to-date or more accurate in a diagnosis.

## Figures and Tables

**Table 1:** General characteristics of respondents.

Variable	n (%)
Age of respondents (years)	
18–24	15 (4.0%)
25–34	252 (67%)
35–44	84 (22%)
45–54	17 (4.5%)
55–64	6 (1.6%)
Gender	
Male	342 (91%)
Female	32 (9%)
Qualifications	
Bachelor of Pharmacy	273 (73%)
Master of Pharmacy	30 (8%)
Doctor of Pharmacy (PharmD)	59 (16%)
PhD	12 (3.2%)
Number of years of practice	
≤1	40 (11%)
1–4	100 (27%)
5–9	100 (27%)
10–14	59 (16%)
>15	33 (8.8%)
Average number of hypertensives seen per month	
<10	26 (7%)
10–20	117 (31%)
>20	231 (62%)
Average number of individuals with diabetes seen per month	
<10	18 (5%)
10–20	92 (25%)
>20	264 (71%)
Average number of smokers seen per month	
<10	52 (14%)
10–20	90 (24%)
>20	232 (62%)

**Table 2:** Correct responses of HDFQ questionnaire.

Questions	n (%)
A person always knows when they have heart disease	209 (56%)
If someone has a family history of heart disease, he/she is at risk for developing heart disease	308 (82%)
The older a person is, the greater their risk of having heart disease	337 (90%)
Smoking is a risk factor for heart disease	364 (97%)
A person who stops smoking will lower their risk of heart disease	349 (93%)
High blood pressure is a risk factor for heart disease	363 (97%)
Keeping blood pressure under control will reduce a person’s risk for developing heart disease	347 (93%)
High cholesterol is a risk factor for developing heart disease	362 (97%)
Eating fatty foods does not affect blood cholesterol	335 (90%)
If someone’s good cholesterol (HDL) is high he/she is at risk for heart disease	297 (79%)
If someone’s bad cholesterol (LDL) is high he/she is at risk for heart disease	348 (93%)
Being overweight increases a person’s risk for heart disease	361 (97%)
Regular physical activity will lower a person’s chance of getting heart disease	356 (95%)
Only exercising at a gym or in an exercise class will lower a person’s chance of developing heart disease	273 (73%)
Walking and gardening are considered exercise that will help lower a person’s chance of developing heart disease	342 (91%)
Diabetes is a risk factor for developing heart disease	329 (88%)
High blood sugar puts a strain on the heart	318 (85%)
If someone’s blood sugar is high over several months it can cause his/her cholesterol level to go up and increase his/her risk of heart disease	308 (82%)
A person who has diabetes can reduce his/her risk of developing heart disease if he/she keeps his/her blood sugar level under control	338 (90%)
Person with diabetes rarely have high cholesterol	284 (76%)
If a person has diabetes, keeping his/her cholesterol under control will help lower his/her chance of having heart disease	339 (91%)
People with diabetes tend to have low HDL (good) cholesterol	196 (52%)
A person who has diabetes can reduce his/her risk of developing heart disease if he/she keeps his/her blood pressure under control	336 (90%)
A person who has diabetes can reduce his/her risk of developing heart disease if he/she keeps his/her weight under control	343 (92%)
Men with diabetes have a higher risk of heart disease than women with diabetes	126 (34%)
Correctly identify examples of cardiovascular disease	193 (52%)
Correctly identify risk factors of cardiovascular disease	263 (70%)

**Table 3:** Knowledge of diagnostic cut-offs for common cardiovascular disease risk factors.

Risk Factor	Cut-Off	n (%)
Hypertension		
	BP > 120/80 mmHg	81 (22%)
	BP > 130/95 mmHg	60 (16%)
	BP ≥ 140/90 mmHg *	184 (49%)
	BP > 150/90 mmHg	49 (13%)
Diabetes		
	FBS ≥ 110 mg/dL	98 (26.2%)
	FBS ≥ 126 mg/dL *	172 (46%)
	FBS ≥ 140 mg/dL	104 (28%)
Abdominal obesity (male)		
	WC > 88 cm	60 (16%)
	WC > 90 cm	98 (26%)
	WC > 102 cm *	216 (58%)
Abdominal obesity (female)		
	WC > 88 cm *	179 (48%)
	WC > 90 cm	101 (27%)
	WC > 102 cm	94 (25%)
Hypercholesterolemia		
	TC ≥ 190 mg/dL	30 (8.0%)
	TC ≥ 200 mg/dL	122 (33%)
	TC ≥ 220 mg/dL	70 (19%)
	TC ≥ 240 mg/dL *	152 (41%)

* Correct answer. BP, blood pressure; FBS, fasting blood sugar; TC, total cholesterol; and WC, waist circumference.

**Table 4:** Association between HDFQ score category and socio-demographic characteristics.

Variable	HDFQ Score		Factors		
	Unsatisfactory, N = 21 ^1^	Satisfactory, N = 353 ^1^	OR ^2^	95% CI ^2^	*p*-Value
Age (years)					
18–24	5 (24%)	10 (2.8%)	Ref	Ref	
25–34	11 (52%)	241 (68%)	13.9	2.44, 79.1	0.003
35–44	2 (9.5%)	82 (23%)	13.0	1.19, 175	0.040
45–54	3 (14%)	14 (4.0%)	1.10	0.08, 14.8	>0.9
55–64	0 (0%)	6 (1.7%)	19,023,662	0.00, NA	>0.9
Sex					
Female	4 (19%)	28 (7.9%)	Ref	Ref	
Male	17 (81%)	325 (92%)	2.08	0.28, 11.9	0.4
Qualifications					
Bachelor of Pharmacy	15 (71%)	258 (73%)	Ref	Ref	
Doctor of Pharmacy (PharmD)	3 (14%)	56 (16%)	1.75	0.40, 11.4	0.5
Master of Pharmacy	1 (4.8%)	29 (8.2%)	2.31	0.30, 62.2	0.5
PhD	2 (9.5%)	10 (2.8%)	0.26	0.05, 2.45	0.2
Number of years of practice					
≤1	4 (19%)	36 (10%)	Ref	Ref	
1–4	7 (33%)	93 (26%)	0.19	0.02, 1.25	0.11
5–9	6 (29%)	136 (39%)	0.37	0.04, 2.85	0.4
10–14	2 (9.5%)	57 (16%)	0.49	0.03, 9.04	0.6
>15	2 (9.5%)	31 (8.8%)	0.56	0.03, 12.9	0.7
Average number of hypertensives seen per month					
<10	5 (24%)	21 (5.9%)	Ref	Ref	
10–20	7 (33%)	110 (31%)	1.75	0.26, 9.77	0.5
>20	9 (43%)	222 (63%)	0.98	0.08, 9.73	>0.9
Average number of individuals with diabetes seen per month					
<10	5 (24%)	13 (3.7%)	Ref	Ref	
10–20	7 (33%)	85 (24%)	4.89	0.77, 33.5	0.094
> 20	9 (43%)	255 (72%)	19.9	1.73, 260	0.019
Average number of smokers seen per month					
<10	5 (24%)	47 (13%)	Ref	Ref	
10–20	5 (24%)	85 (24%)	0.78	0.14, 3.95	0.8
>20	11 (52%)	221 (63%)	0.57	0.11, 2.39	0.5

^1^ n (%). ^2^ OR = Odds Ratio, and CI = Confidence Interval.

## Data Availability

The data used to analyze this survey will be provided by the corresponding author upon request.
